# Influence of the Severity of Infective Endocarditis on Long-Term Outcomes After Mitral Valve Surgery

**DOI:** 10.7759/cureus.87934

**Published:** 2025-07-14

**Authors:** Tadashi Omoto, Atsushi Aoki, Kazuto Maruta, Akitoshi Takazawa, Tomoaki Masuda

**Affiliations:** 1 Cardiovascular Surgery, Showa Medical University, Tokyo, JPN; 2 Cardiovascular Surgery, Kawasaki Municipal Hospital, Kawasaki, JPN

**Keywords:** infective endocarditis, long-term outcome, mitral regurgitation (mr), mitral valve surgery, scores

## Abstract

Objectives: Previous studies have demonstrated the advantages of mitral valve repair (MVP) in infective endocarditis (IE) in early and late outcomes. However, the influence of complexity in mitral valve surgery on long-term outcome is unclear. The purpose of this study was to clarify whether the severity of IE affects long-term outcome.

Methodology: Fifty-one patients who underwent mitral valve surgery during the active phase of native IE were retrospectively reviewed. Severity score was used as an index for the severity of IE. Severity score assigns a score derived from two aspects: (1) the extent of valvular destruction and (2) technical difficulties for predicting the feasibility of MVP reported by our previous studies. Patient profile, long-term survival, and freedom from recurrence of mitral regurgitation (MR) were compared between those with a severity score of 7 or less (*low score*: *n* = 17) and with a severity score of 8 or more (*high score*: *n* = 34). Recurrence of MR was defined as a more than moderate grade of MR.

Results: Body weight was lower and body surface area was smaller in *high score*, and concomitant aortic valve operation was more frequently performed in *high score *than in *low score*. MVP was more frequently performed in *low score* than in *high score* (88% vs. 38%, *P *< 0.001), and patients in *low score *showed a better 10-year survival rate than those in* high score* (100% vs. 70%; log-rank, *P *= 0.020). Freedom from recurrence of MR in 10 years after MVP was 100% vs. 53% in *low score* and *high score*, respectively (*P *= 0.008).

Conclusions: Severity of IE is associated with long-term outcome. Patients with low severity have high feasibility of MVP, low risks of reoperation, and good long-term survival. In patients with high severity, the indication for complex repair should be considered, taking age and future redo surgery into consideration.

## Introduction

It has been demonstrated that mitral valve repair (MVP) for active infective endocarditis (IE) offers superior early and late outcomes compared with mitral valve replacement (MVR), and the advantage has been explained by preservation of left ventricular function or avoidance of prosthetic valve which has potential risk of recurrence of IE and paravalvular leakage [1-3]. However, these previous studies were derived from data based on small sample sizes and were unable to compare with each other because of their heterogeneity in operative indication, repair experiences, or repair techniques. Furthermore, it has been controversial whether poor surgical outcomes of MVR result from poor preoperative conditions.

The probability of MVP might be associated with the severity of IE because a severely damaged leaflet is difficult to repair and requires to be replaced with a prosthetic valve. To assess the difficulties in MVP for degenerative mitral valve disease, complexity score was advocated by Mount Sinai Hospital, assigning a score to each valve based on the following: prolapsing segments (weight 1 for each posterior segment; weight 2 for each anterior and commissural segment); presence of valve restriction (weight 2); presence of calcification (weight 3 if annulus involved, otherwise weight 2); and prior mitral valve repair (weight 3) [4]. To assess the probability of MVP for patients with IE, we have developed a severity score by (1) the extensiveness of leaflet destruction, which is similar to the complexity score, and additionally, (2) the complexity of repair technique (Appendix) [5]. By retrospective review of each surgical case, the probability of feasible MVP could be categorized into high, moderate, and low probability according to severity score as 7 points or less, 8 points, and 9 points or more [5].

The purpose of this study was to clarify the relationship between the complexity of MVP and long-term outcome in patients with active IE undergoing mitral valve surgery.

## Materials and methods

The protocol for this retrospective study was approved by the Institutional Review Board of Showa Medical University (IRB No. 3123 approved on May 7, 2022). Patients have been given opt-out information regarding this study.

Between December 2004 and July 2023, 124 patients with active IE who underwent surgical intervention were retrospectively reviewed. One patient opted out, and 51 patients with native mitral valve endocarditis were included in the analysis. The requirement for informed consent was waived owing to the retrospective study design. If surgical intervention was required before completion of a standard course of intravenous antibiotics, IE was defined as active IE [5,6]. The most suitable antimicrobial regimen according to the guideline [7] was continued four to eight weeks postoperatively until serum C-reactive protein (CRP) decreased below 1 mg/dL.

The severity score was used as an index of IE severity. It was assessed intraoperatively using transesophageal echocardiography, with the final determination made after resection of all infective valvular and subvalvular lesions. Reviewed patients were divided into two groups: those with a severity score of 7 or less (low-score group: *n* = 17) and those with a severity score of 8 or more (high-score group: *n* = 34). Survival and cardiac events, including relapse of endocarditis or recurrent MR after discharge, were reviewed by clinical records at the outpatient clinic or by contacting the patient’s general practitioners and compared between the two groups.

Major complication was defined as cerebrovascular (cerebral infarction, intracranial bleeding), cardiac (myocardial infarction, heart failure, left ventricular rupture), respiratory (pneumonia, respiratory failure), gastrointestinal complications (gastrointestinal bleeding, intraperitoneal infection, mesenteric ischemia), and new-onset renal failure requiring renal replacement therapy. Mitral regurgitation (MR) was graded by color-flow and pulsed-wave Doppler echocardiography as none, mild, moderate, and severe [8], and more than moderate postoperative MR was defined as recurrent MR.

The Japan score was used as a risk score to predict perioperative mortality [9,10]. The Society of Thoracic Surgeons (STS) score and the European System for Cardiac Operative Risk Evaluation (EuroSCORE) II are more familiar; however, their application to Japanese patients might be inappropriate due to racial differences. The Japan score has been developed to establish an original Japanese risk predictive model. For example, the operative risk of patients undergoing aortic valve replacement for aortic stenosis was 3.1%, 4.9%, and 3.2% by EuroSCORE, STS score, and Japan score [10]. Risk factors such as emergency surgery, preoperative creatine level >3.0 mg/dL, aortic valve stenosis, or obstructive lung disease showed high predictive accuracy by the Japan score [9,10].

Statistical analysis

Statistical analysis was performed using JMP Pro 16 (SAS Inc., Cary, NC), and continuous variables were expressed as mean with standard deviation. Categorical variables were expressed as a proportion (%). Assessment of differences in baseline characteristics and preoperative patient profiles in each of the three categories was performed using Wilcoxon analysis, and χ^2^ analysis or Fisher’s exact test for categorical variables.

Survival and freedom from recurrent MR over time were analyzed using Kaplan-Meier methods, and differences between survival curves were tested with the log-rank χ^2^ statistic. *P*-values < 0.05 were considered statistically significant.

## Results

Patient profiles and operative findings

The preoperative characteristics of patients are presented in Table 1.

**Table 1:** Preoperative patient profile. *P < 0.05. BSA, body surface area; pre-CRP, preoperative serum c-reactive protein; pre-CI, preoperative cerebral infarction; pre-HF, preoperative heart failure; AV, aortic valve involvement; HD, hemodialysis

	Low score (*n* = 17)	High score (*n *= 34)	*P*-value
Age (years)	58.0 ± 13.2	61.3 ± 17.9	0.313
Female, *n* (%)	4 (24%)	13 (38%)	0.358
Height (cm)	166.3 ± 2.2	162.8 ± 1.6	0.342
Weight (kg)	62.0 ± 8.6	55.1 ± 10.1	0.026*
BSA	1.68 ± 0.13	1.58 ± 0.16	0.022*
Pre-CRP (mg/dL)	3.8 ± 3.4	5.6 ± 5.4	0.251
Pre-Alb (mg/dL)	2.6 ± 0.7	2.7 ± 0.6	0.772
DM, *n* (%)	4 (24%)	4 (24%)	0.276
Causative microorganisms, *n* (%)			
Streptococcus species	8 (47%)	19 (55%)	0.552
Staphylococcus aureus	6 (35%)	6 (18%)	0.168
Preop days	24.2 ± 19.5	13.2 ± 9.8	0.067
Japan Score	5.7 ± 4.8	10.0 ± 11.1	0.077
Japan Score of 8 or more than 8	3 (19%)	15 (46%)	0.065
Embolic event	7 (41%)	10 (29%)	0.401
Pre-CI	7 (41%)	8 (24%)	0.192
Pre-HF	5 (29%)	16 (47%)	0.227
AV	3 (19%)	16 (47%)	0.041*
HD	1 (6%)	2 (6%)	0.999

Body weight and body surface area were lower in the high-score group than in the low-score group. The duration of preoperative antibiotic therapy tended to be shorter in the high-score group than in the low-score group. Aortic valve involvement was more frequent in the high-score group.

Causative microorganisms

Causative microorganisms were isolated and identified in blood cultures in 50 patients, except for one patient. The microorganisms responsible for endocarditis were Streptococcus species in 29 patients, Staphylococcus aureus in 11 (including 2 patients with methicillin-resistant Staphylococcus aureus), Enterococcus faecalis in 6, and others, including Escherichia coli, Cardiobacterium hominis, Gemella haemolysans, and Granulicatella adiacens, in one case each. There were no differences in the frequency of Streptococcus species or Staphylococcus aureus between the high-score and low-score groups.

Embolic events

A total of 21 preoperative embolic events were identified in 18 patients (cerebral: 13; splenic: 3; renal: 2; ocular: 2; peripheral: 1). There were no differences in their occurrence between the two groups (Table 1).

Operative profiles and early outcomes 

Operative profiles are shown in Table 2.

**Table 2:** Operative profile and early outcomes. *P < 0.05. MVP, mitral valve repair; ACC, aortic cross-clamp time; CPB, cardiopulmonary bypass time

	Low score (*n* = 17)	High score (*n* = 34)	*P*-value
MVP	15 (88%)	13 (38%)	<0.001*
Patch reconstruction	2 ((13%)	12 (92%)	<0.0001*
Artificial chordae	3 (20%)	7 (54%)	0.062
ACC (min)	111 ± 29	125 ± 43	0.339
CPB (min)	148 ± 44	158 ± 49	0.442
Major complication	5 (29%)	14 (44%)	0.413
In-hospital mortality	3 (18%)	4 (12%)	0.565

There were no differences in cardiopulmonary bypass time and aortic cross-clamp time between the two groups. Ring annuloplasty was performed in all patients undergoing MVP, including an autologous pericardial ring in each case in both groups. There was a greater tendency to use artificial chordae in the high-score group than in the low-score group, and autologous pericardial patch reconstruction was used more frequently in the high-score group. All patients who underwent MVP left the operating room with an MR grade less than mild: 4 patients were in mild grade, and 24 patients were in trivial grade.

There were no differences in the rate of major complications and in-hospital mortality between the two groups (Table 2). There were two in-hospital deaths in the low-score group (peritonitis: 1; respiratory failure: 1) and one in the high-score group (respiratory failure).

Long-term survival in low-score and high-score groups

The completeness of follow-up was 100% in reviewed patients who were discharged (*n* = 25), and the median follow-up time was 123 months (1-214 months). Follow-up physicians belonged to our institute (*n* = 11), referral general hospital (*n *= 10), and general practitioners (*n *= 4). Four patients who regularly visited general practitioners had not undergone recent transthoracic echocardiography; however, there was no apparent systolic regurgitation on apical auscultation, nor was there an increased cardiothoracic ratio on chest X-ray. The other 21 patients underwent transthoracic echocardiography every one to three years.

**Figure 1:**
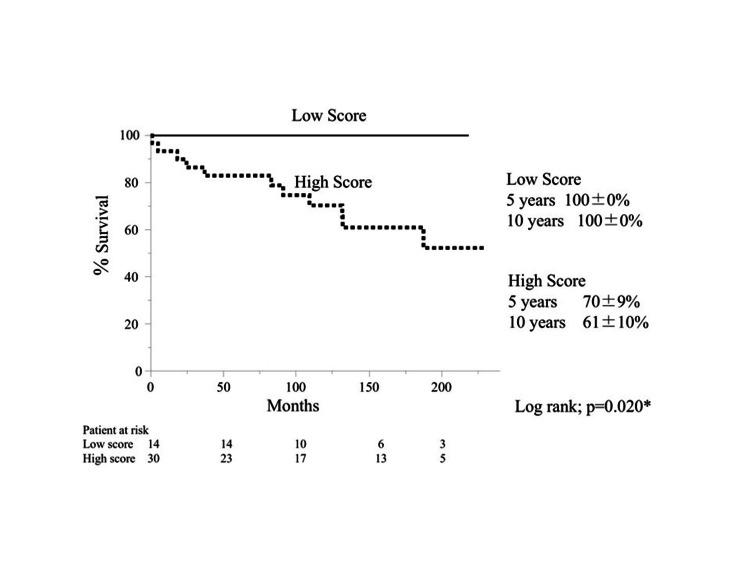
Kaplan-Meier actuarial survival curve: high vs. low scores.

The actuarial survival curve is shown in Figure 1.

There was no difference between patients in *high score* and *low score* in 5- and 10-year survival rates (93% vs. 100%, and 71% vs. 100%, respectively; log-rank: *P *= 0.207).

**Figure 2:**
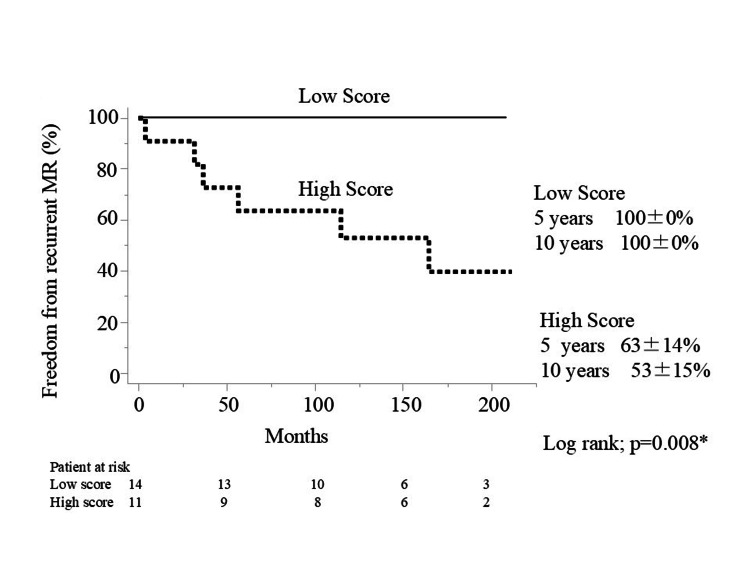
Freedom from recurrence of mitral regurgitation.

Freedom from recurrent MR at 5 and 10 years after MVP was 100% vs. 63%, and 100% vs. 53% in the low-score and high-score groups, respectively (*P* = 0.008) (Figure 2).

No patients in the low-score group developed more than moderate MR. In contrast, six patients in the high-score group developed recurrent MR (Table 3).

**Table 3:** Patient with recurrent mitral regurgitation. M, month; Y, year; MVR, mitral valve replacement; MR, mitral regurgitation; PML, posterior mitral leaflet; AML, anterior mitral leaflet; HF, heart failure

Case	Age/Gender	Lesion	Procedure	Event	Alive/Death
1	60, male	A2-3, PC, P2-3	Patch repair: A3-P3, artificial chordae	6M: redo MVR	Alive
2	48, male	A2-3	Patch repair: A2-3, artificial chordae	10Y: redo MVR	Alive
3	33, male	A3-PC	Patch repair A3-PC, artificial chordae	14Y: redo MVR	Alive
4	51, male	A2	Patch repair A2	10Y: redo MVR	Alive
5	13, male	A3-PC-P3	Patch repair: A3-P3	3M~: mild to moderate MR, conservative	Alive
6	64, male	Restrictive PML	Patch: AML, artificial chordae	10Y: severe MR, refused operation	Dead/HF

Out of six patients who developed recurrent MR, four patients underwent redo MVR successfully (Cases 1-4; Table 3). The cause of MR was degenerative changes in the autologous pericardial patch in three cases and suture dehiscence in one case. A 13-year-old male patient (Case 5; Table 3) who underwent MVP with patch reconstruction covering A3-P3 developed mild to moderate MR three months after surgery; however, the condition did not progress to a severe grade and was managed medically without clinical signs of heart failure for 14 years postoperatively. A 64-year-old male patient (Case 6; Table 3) who underwent MVP with patch reconstruction covering A1-A2 and P3, along with artificial chordae supporting A1 and A2, developed mild to moderate MR seven months after the operation. The severity of MR gradually progressed to more than moderate grade nine years postoperatively. He was recommended for redo surgery; however, he refused to undergo further invasive therapy. He died 10 years after the operation due to congestive heart failure.

In the subgroup analysis of high-score patients comparing MVP and MVR (Group II vs. Group III; Table 4), patients who underwent MVP were younger, had higher preoperative serum albumin levels, were less likely to have a Japan score of 8 or higher, and had a shorter duration of preoperative antibiotic therapy compared to those who underwent MVR.

**Table 4:** Patient profile of three subgroups. *P < 0.05. MVP, mitral valve repair; MVR, mitral valve replacement; BSA, body surface area; Pre-CRP, preoperative serum C-reactive protein; Pre-CI, preoperative cerebral infarction; Pre-HF, preoperative heart failure; AV, aortic valve involvement; HD, hemodialysis

Subgroup	Group I: MVP/Low score (*n* = 15)	Group II: MVP/High score (*n* = 13)	Group III: MVR/High score (*n* = 21)	*P*-value: I vs. II	*P*-value: II vs. III
Age (years)	56.6 ± 13.4	48.9 ± 19.1	68.9 ± 12.1	0.299	0.002*
Female, *n* (%)	3 (20%)	2 (15%)	11 (52%)	0.751	0.025*
Height (cm)	167 ± 10	167 ± 6.0	160 ± 9.2	0.982	0.029*
Weight (kg)	62.7 ± 8.8	54.2 ± 8.2	55.6 ± 11.2	0.021*	0.632
BSA	1.7 ± 0.1	1.6 ± 0.1	1.6 ± 0.2	0.072	0.583
Pre-CRP (mg/dL)	3.5 ± 3.5	4.3 ± 2.7	6.4 ± 6.6	0.279	0.697
Pre-Alb (mg/dL)	2.7 ± 0.8	3.1 ± 0.3	2.5 ± 0.6	0.253	0.026*
DM	4 (27%)	1 (8%)	3 (14%)	0.191	0.935
Staphylococcus aureus	5 (33%)	1 (8%)	5 (24%)	0.502	0.208
Preop days	25.9 ± 20.1	9.0 ± 7.0	15.8 ± 10.5	0.021*	0.057
Japan score	5.3 ± 4.9	5.2 ± 3.6	12.2 ± 12.9	0.908	0.015*
Japan scores of 8 or more than 8	3 (20%)	3 (23%)	12 (57%)	0.843	0.047
Pre-CI	6 (40%)	2 (15%)	6 (29%)	0.143	0.378
Pre-HF	4 (27%)	3 (23%)	13 (62%)	0.823	0.025*
AV	2 (13%)	5 (38%)	11 (52%)	0.123	0.427
HD	0 (0%)	0 (0%)	2 (9.5%)	0.999	0.251

**Figure 3:**
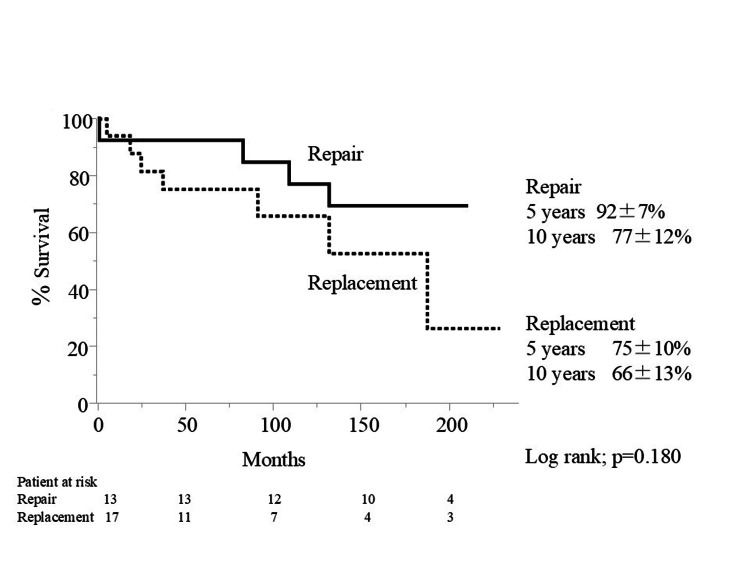
Actuarial survival rate in the high-score group.

There was no difference in 5- and 10-year survival rates between patients undergoing MVP and MVR in the high-score group (Figure 3).

Redo surgeries and late deaths in the high-score group are summarized in Table 5.

**Table 5:** Redo surgeries and late deaths in the high-score group.

Late events	MVP (High score) (*n* = 13)	MVR (High score) (*n* = 17)	*P*-value
Redo	4 (31%)	2 (12%)	0.197
Late death	4 (31%)	7 (41%)	
	Heart failure (1)	Heart failure (2)	
	Malignancy (1)	Respiratory failure (3)	
	Unknown (2)	Malignancy (2)	

A total of six redo operations were performed in the reviewed cases. In all six, no causal relationship to infection was identified. Two cases following MVR required repeat mitral valve replacement due to structural valve destruction and paravalvular leakage. Postoperative courses were uneventful in both cases for 11 and 13 years, respectively. The four cases following MVP are described in Table 3. 

## Discussion

Previous studies have demonstrated that MVP in active IE is associated with better long-term survival compared with MVR [1-3]. Ruttmann et al. studied 68 patients with mitral valve endocarditis in the active phase and showed that better early and late results [1]. Feringa et al. systematically reviewed 24 studies, including 470 patients who underwent MVP or MVR. Of the 470 patients who underwent MVP, 33 patients died (7%), as compared with 241 patients (33%) who underwent MVR (*P *< 0.0001) [2]. Toyota et al. studied 1,970 patients in New York and California, and patients undergoing MVP showed better 12-year survival than MVR (69% vs. 54%, *P *= 0.002) [11]. These superior outcomes have been explained by the avoidance of a prosthetic valve, which carries a potential risk of recurrent IE and paravalvular leakage, in addition to the generally recognized advantages of MVP, such as superior ventricular function through preservation of the subvalvular apparatus [1-3]. Potential risk of infection of prosthetic valve may be explained by blood flow turbulence caused by intracardiac structural abnormality, resulting in endothelial disruption and platelet and fibrin deposition serving as a nidus for subsequent adhesion by bacteria.

Poor long-term outcomes in *high scores* may be due to a lower probability of MVP. This study demonstrated that those who have a low severity of endocarditis have a high feasibility of MVP and showed good long-term outcomes. And also, the MVP in patients with low severity showed better long-term durability than those with high severity. There is an impact of the complexity of repair on long-term durability, and repair with patch reconstruction after extensive leaflet resection has less durability than simple leaflet resection and suture technique. 

Echocardiography plays a decisive role in the diagnosis of IE as well as the treatment strategy. A major criterion of modified Duke criteria is the demonstration of endocardial involvement with vegetations, paravalvular extension of infection, or evidence of disruption of the valve by echocardiography. To define the severity score, the following assessments are required from intraoperative transesophageal echocardiography: (1) characteristics of the vegetation, such as size, mobility, and whether it is sessile or pedunculated; (2) location of the vegetation to determine the valvular score; and (3) identification of the regurgitation mechanism to estimate which chordae and associated leaflet can be preserved. Three-dimensional echocardiography appears to provide improved visualization of valve structure and function for defining the severity score.

**Figure 4:**
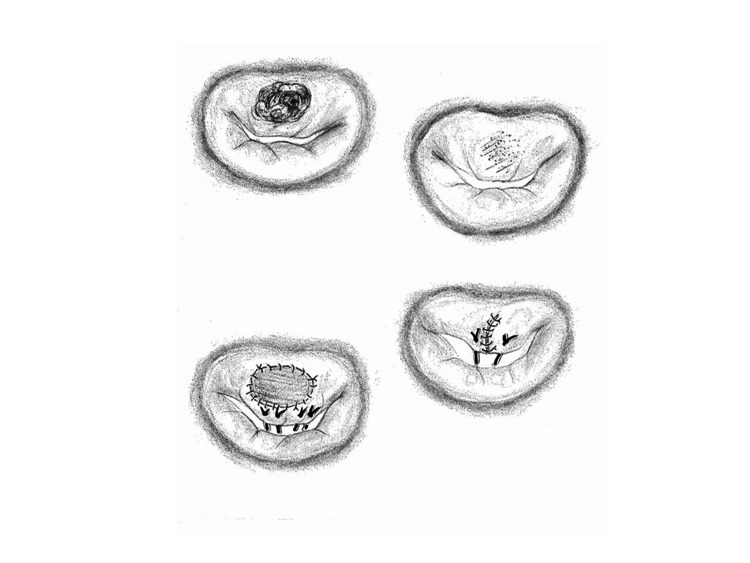
Three types of operative procedures for an A2 lesion. Three types of operative procedures for an A2 lesion are shown: upper left – classification overview; upper right: a case without leaflet defect (severity score = 2); lower right: a case with leaflet closure (severity score = 2+3); lower left: a case with patch reconstruction (severity score = 2+3+3). Image credit: All authors.

The severity scoring system is based on the suture stress of the reconstructed leaflets, which influences long-term durability. For example, suture stress of the anterior mitral leaflet is supposed to be higher than the posterior mitral leaflet, and the A2 scallop is assumed to have the highest mobility. An A2 lesion can be categorized into three types based on the severity score (Figure 4): (1) a case without leaflet defect after vegetation removal (valvular score = 2, technical score = 0); (2) a case with a leaflet defect requiring direct closure (valvular score = 2, technical score = 3); or (3) a case requiring patch reconstruction (valvular score = 2, technical score = 3+3). 

The following two cases serve as representative examples of moderate probability for MVP (severity score = 8), involving large patch reconstruction in: (1) the A2 scallop, or (2) the commissural leaflet, affecting both the anterior and posterior leaflets. If the assessment by intraoperative transesophageal echocardiography shows less severity than those two typical cases, MVP will be expected to be highly feasible, and if the assessment is more severe than those two cases, MVP will be carefully selected, taking age and operative risk into consideration.

The first priority of surgical strategy against IE is the avoidance of early mortality. We have previously studied the operative timing and feasibility of MVP in patients undergoing acute phase surgery and demonstrated that surgery within 48 hours resulted in high morbidity and mortality [6]. Although it is difficult to draw a clear conclusion regarding operative timing of mitral valve surgery during the active phase of IE, it might be within 48 hours to 14 days in light of early operative results and severity of mitral leaflet destruction [6]. Our results may suggest that the operative schedule could be delayed in patients with a low severity score, and earlier intervention might be considered in patients with a high severity score. 

There are some limitations of this study. As this is a retrospective study based on clinical experience at a single institution with a small sample size, the ability to draw definitive conclusions is limited. However, collecting information about patient survival, mortality, and the cause of death was highly accessible due to a single-center experience. The severity score was based on surgical outcomes by our institute, and the arbitrary nature might be inevitable. Although there were no recurrences of endocarditis in the reviewed patients, subclinical re-infection events might have developed without diagnosis and resulted in recurrent MR. In some patients in our series, an echocardiographic follow-up was not able to be performed. In order to detect subclinical re-infection, which may develop into definitive IE, routine blood examinations, including leukocyte count and CRP, echocardiography, and, most of all, the elicitation of fever episodes should not be overemphasized.

## Conclusions

Severity of IE is associated with long-term outcomes. Patients with low severity scores showed good long-term outcomes and freedom from recurrence of MR. In patients with high severity scores, MVP was performed in mild cases with a good prognosis. In severe cases, prognosis might be better with MVP than with MVR, but reoperation was supposed to be more common. Assessment of the severity score by intraoperative transesophageal echocardiography plays an important role in operative decision-making and predicting prognosis.

## Appendices

Appendix 

**Table 6:** Severity score. Taken from [5].

Valvular Score	Weight
P1-P3	1
Commissure leaflet (anterior/posterior)	1
A1-A3	2
Preexisting lesion (restrictive or rheumatic)	2
Abscess	2
Technical Score	
Leaflet resection/ one scallop	1
Leaflet resection/ two scallop	2
Leaflet resection/ A2	3
Patch reconstruction/ one scallop	1
Patch reconstruction/ two scallop	2
Patch reconstruction/A2	3
